# Investigation on the Genetic Signatures of Antibiotic Resistance in Multi-Drug-Resistant Klebsiella Pneumoniae Isolates From National Guard Hospital, Riyadh

**DOI:** 10.7759/cureus.11288

**Published:** 2020-11-01

**Authors:** Hassan N Khdary, Abdullah Almalki, Mohamad H Alkhdiri, Saad Alhamoudi, Abdullah Alfaleh, Majed F Alghoribi, Taher Uz Zaman

**Affiliations:** 1 Infectious Disease, King Saud Bin Abdulaziz University for Health Sciences College of Medicine, Riyadh, SAU; 2 Infectious Disease, King Abdullah International Medical Research Center, Riyadh, SAU

**Keywords:** klebsiella pneumoniae (kp), resistance genes, multi-locus sequence typing (mlst)

## Abstract

Introduction

Despite a large number of antibiotics available to treat Klebsiella (K.) pneumoniae (KP), resistance against these antibiotics is ever-increasing and has now become a global threat to human life. The most frequently observed resistant genes in Klebsiella pneumoniae are CTX-M, OXA-48, IMP, and NDM; some are clone-specific while others form a reservoir for infection.

Methods

Matrix-assisted laser desorption ionization-time of flight (MALDI-TOF) was employed for the identification of the pathogens and automated VITEK-2 (bioMérieux, Marcy-l'Étoile, France) was used for minimum inhibitory concentration (MIC) determination, followed by polymerase chain reaction (PCR) amplification of target genes and Sanger sequencing of amplicons.

Results

Forty-three out of 50 isolates (86%) were OXA gene-positive, and 49 out of 50 (98%) isolates were CTX-M gene positive. Two phenotypes of OXA were identified in 33 samples sequenced, OXA-505 (70%) and OXA-232 (30%). Sixteen isolates (32%) were positive for NDM-1. Twelve isolates were positive for both OXA and NDM. Multilocus sequence typing (MLST) on these isolates showed that they were distributed in 12 sequence types (STs). Thirty-six out of 50 were grouped in four clonal complexes. ST-14 was the predominant genotype.

Conclusion

This study has revealed that CTX-M-15 is the most common extended-spectrum beta-lactamase (ESBL) present in almost all isolates. The study also shows the presence of OXA as the main carbapenemase gene, alone or in combination with other carbapenemases such as NDM-1. Multilocus sequence typing revealed the incidence of polyclonal KP pool with ST-14, ST-29, ST-307, and ST-15 being the predominant ones.

## Introduction

Resistance against antibiotics is ever-increasing and has now become a global threat to human life [1-2]. Bacterial pathogens have developed resistance against many of these antibiotics, and some countries have reported strains that are resistant to all antibiotics available, i.e. pan-drug resistance [3]. Various mechanisms involving a battery of diversified genes are described for these resistances. Some of these genes are found on the bacterial chromosome, whereas others are found on episomal deoxyribonucleic acid (DNA) fragments called plasmids [4]. These plasmid-mediated genes can transfer resistance horizontally from one bacterium to another by inter-species or intra-species conjugations. The most frequently observed resistant genes in Klebsiella (K.) pneumoniae (KP) are TEM, SHV, CTX-M, OXA-48, IMP, VIM, NDM, and KPC. Some are clone-specific while others are transferred independently. Carbapenems are supposed to be the last line of drugs available against these pathogens. However, carbapenems have now also been reported to be ineffective due to the emergence of new resistant genes, such as KPC, OXA, NDM, VIM, and IMP, against this antibiotic [5]. Although the KPC gene is not much prevalent in Saudi Arabia, OXA-48 is widely reported in carbapenem-resistant Klebsiella pneumonia [6]. The New Delhi metallo-beta-lactamase 1 (NDM-1) gene is also being reported regularly from different parts of Saudi Arabia [7-8]. Few studies are available on the molecular basis of antibiotic resistance, virulence factors present, plasmid profiles carrying resistance genes, and the clonal diversity of these drug-resistant Klebsiella pneumoniae in Saudi Arabia. However, the true picture of antibiotic resistance in Klebsiella pneumoniae from the Kingdom in general and the National Guards Hospital, Riyadh, in particular still remains unclear. The aim of the present research is to understand the mechanisms of antibiotic resistance in multi-drug Carbapenem-resistant isolates of Klebsiella pneumoniae from the national guard hospital by investigating the genetic signatures of these resistant genes. We would also determine the genotypes of these strains that are predominant in this region to facilitate the control programs. All of this could help improve the infection control intervention and promote/support effective antibiotic stewardship programs in the National Guard Hospital, Saudi Arabia.

## Materials and methods

The study was conducted at the infectious diseases laboratory of King Abdullah International Medical Research Center (KAIMRC). Matrix-assisted laser desorption ionization-time of flight (MALDI-TOF) was employed for the identification of the pathogens and automated VITEK-2 (bioMérieux, Marcy-l'Étoile, France) was used for minimum inhibitory concentration (MIC) determination. The isolates, which showed elevated MIC for carbapenem and beta-lactam antibiotics and were designated as resistant by the microbiology department, National Guard Hospital, were the subject of this present study. Detection of resistance gene targets, such as CTX-M, OXA-48, and NDM, was done by polymerase chain reaction (PCR) using their specific primers. The sequencing of the gene products was done by the Sanger method to look for their variants (novel, if any). Multilocus sequence typing (MLST) was performed on these isolates using seven loci scheme proposed by Diancourt et al. [9] to determine their clonality and sequence type (ST).

Data collection process

Bacterial isolates were derived from patients on routine lab investigations. The sources of these isolates are given in Table 1. The identification was done by the MALDI-TOF technique in the microbiology lab. MICs were determined by VITEK-2. The KP isolates with elevated MIC were grown on blood agar medium. A single colony was selected and grown in a liquid broth medium. Fifty isolates of multi-drug carbapenem-resistance of Klebsiella pneumonia were investigated. DNA was extracted and PCR amplification of genes of antibiotic resistance (CTX-M, NDM, OXA) was done using the primers specific for these genes. These amplified products were visualized on an agarose gel by electrophoresis. Positive and negative controls were included. The confirmed positive PCR products were processed for sequencing by the Sanger Sequencing method. The DNA sequences were aligned and analyzed using Lasergene software. Mega 5 software was used for detecting single nucleotide polymorphisms (SNPs) and for phylogenetic analysis [10]. Each sequence was blasted against either the National Center for Biotechnology Information (NCBI) gene bank or a specific database for phenotype designation of these genes. The various phenotypes of the resistance genes of CTX-M, NDM, and OXA were determined and reported. Five researchers worked on the data collection process and the study took two years.

## Results

Demography

The sources of these isolates are given in Table 1.

**Table 1:** The demographic details and distribution of clonal types and antibiotic-resistant genes

Isolate	Sequence type	Sampling Date	Specimen	Carbapenemase gene	Others
RD-231	14	6/01/2015	respiratory	OXA-232 + NDM	CTX-M
RD-232	15	10/01/2015	urine	NDM	CTX-M
RD-233	NA	13/01/2015	respiratory	OXA (phenotype NA)	CTX-M
RD-234	15	18/01/2015	wound	OXA-505	CTX-M
RD-235	22	23/01/2015	wound	Negative	CTX-M
RD-236	29	23/01/2015	wound	OXA (phenotype NA)	CTX-M
RD-237	NA	25/01/2015	respiratory	OXA-505	CTX-M
RD-238	152	6/02/2015	urine	OXA-505 + NDM	CTX-M
RD-239	14	6/02/2015	urine	OXA + NDM	CTX-M
RD-240	29	8/02/2015	respiratory	OXA-505	CTX-M
RD-241	15	12/02/2015	QC	OXA (phenotype NA)	CTX-M
RD-242	29	12/02/2015	respiratory	OXA-505	CTX-M
RD-243	211	12/02/2015	wound	OXA-505	CTX-M
RD-244	29	21/02/2015	respiratory	OXA-505	CTX-M
RD-245	NA	10/03/2015	respiratory	OXA-505	CTX-M
RD-246	29	12/03/2015	blood	OXA (phenotype NA)	CTX-M
RD-247	14	17/03/2015	respiratory	OXA-232 + NDM	CTX-M
RD-248	29	27/03/2015	wound	OXA-505	CTX-M
RD-249	14	2/04/2015	respiratory	OXA-232	CTX-M
RD-250	15	15/04/2015	urine	Negative	CTX-M
RD-251	NA	18/04/2015	wound	NDM	Negative
RD-252	NA	21/04/2015	urine	OXA-505	CTX-M
RD-253	716	22/04/2015	blood	OXA-505	CTX-M
RD-254	15	27/04/2015	blood	OXA-505	CTX-M
RD-255	NA	29/04/2015	blood	NDM	CTX-M
RD-256	NA	1/05/2015	wound	OXA (phenotype NA)	CTX-M
RD-257	NA	6/05/2015	blood	OXA (phenotype NA)	CTX-M
RD-258	NA	5/05/2015	blood	OXA-505	CTX-M
RD-259	307	9/05/2015	urine	OXA-505	CTX-M
RD-260	14	12/05/2015	wound	OXA-232 + NDM	CTX-M
RD-261	14	15/05/2015	urine	OXA-232 + NDM	CTX-M
RD-262	14	17/05/2015	urine	OXA-232	CTX-M
RD-263	NA	13/05/2015	wound	OXA (phenotype NA)	CTX-M
RD-264	11	22/05/2015	others	NDM	CTX-M
RD-265	14	10/05/2015	blood	OXA-232 + NDM	CTX-M
RD-266	NA	13/05/2015	blood	OXA (phenotype NA)	CTX-M
RD-267	485	30/05/2015	urine	OXA-505	CTX-M
RD-268	14	3/06/2015	urine	OXA-232	CTX-M
RD-269	14	10/06/2015	blood	OXA + NDM	CTX-M
RD-270	17	12/06/2015	Respiratory	OXA-505	CTX-M
RD-271	14	11/06/2015	blood	OXA-505 + NDM	CTX-M
RD-272	1399	17/06/2015	wound	OXA-505	CTX-M
RD-273	307	18/06/2015	blood	OXA-505	CTX-M
RD-274	307	19/06/2015	blood	NDM	CTX-M
RD-275	716	1/07/2015	blood	OXA-505	CTX-M
RD-276	NA	1/07/2015	urine	OXA-505 + NDM	CTX-M
RD-277	14	2/07/2015	urine	OXA-232 + NDM	CTX-M
RD-278	307	3/07/2015	blood	OXA-505	CTX-M
RD-279	14	3/07/2015	urine	OXA-505	CTX-M
RD-280	14	3/07/2015	urine	OXA-232 + NDM	CTX-M

The clinical sources of the specimen for isolation of KP was as follows: respiratory (n = 10); surgical wound (n = 10); QC (n = 1); urine (n = 14); blood (n = 14); and Others (n = 1).

Multilocus sequence typing

The multi-locus sequence typing (MLST) method using seven loci) showed 38 isolates being distributed in 12 STs. Twelve isolates could not be typed due to technical reasons. The distribution of STs was as follows: ST-14 (n=14), ST-15 (n=5), ST-29 (n=6), ST-307 (n=4), ST-716 (n=2), ST-152 (n=1), ST-22 (n=1), ST-211 (n=1), ST-185 (n=1), ST-17 (n=1), ST-485 (n=1), and ST-1399 (n=1). ST-14 was the most common, with 14 isolates (28%), followed by ST-29, ST-15, ST-307, and ST-716.

**Table 2:** MLST complexes MLST: multilocus sequence typing

MLST	n	CG	GapA	infB	mdh	pgi	phoE	rpoB	tonB
14	12	C1	1	6	1	1	1	1	1
15	7		1	1	1	1	1	1	1
22	1	C2	2	3	1	1	1	4	4
29	6		2	3	2	2	1	4	4
152	1		2	3	2	1	1	4	56
105	1		2	3	2	1	1	4	18
10002	1	C3	3	3	6	1	7	4	38
10003	1		3	3	1	1	9	4	43
10006	1		3	3	1	1	1	4	31
10007	1	C4	2	5	1	1	7	1	15
2648	2		2	5	1	1	7	1	6
4698	2		2	5	1	2	7	1	10

Resistance genes profile

All isolates were resistant to more than three types of antibiotics for MIC. The antibiogram profile of these isolates is given in Table 1.

**Figure 1:**
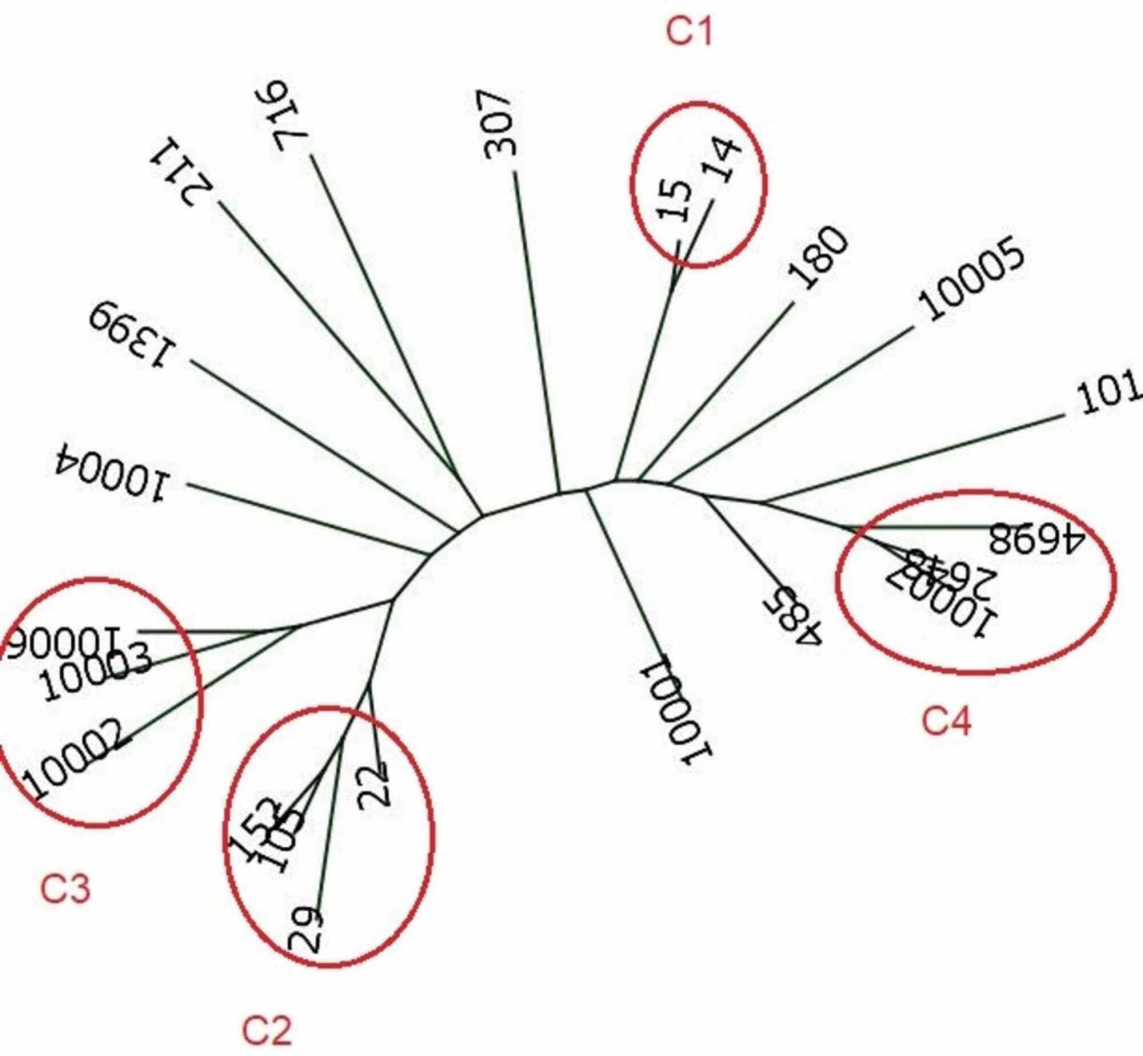
Phylogenic tree

All isolates were resistant to more than three types of antibiotics. CTX-M was the most prevalent, with 49 out of 50 (98%) positive samples, and was carried by isolates of all STs. All the representative 28 samples sequenced were CTX-15. The OXA gene was the most common carbapenemase gene. Sequence analysis of the ~ 740-base pair PCR product revealed an OXA gene allele sensu stricto in all the positive isolates. Forty-three out of 50 (86%) of the samples were positive for OXA. Twenty-three out of 33 sequenced samples (70%) were OXA-505 while 10 (30%) were OXA-232. The phenotypes of the remaining OXA positive isolates could not be determined because of some technical reasons. Twenty-eight out of 50 of the samples (56%) were positive for NDM. The seven amplicons that were subjected to sequencing turned out to be NDM-1. No other allelic form of the NDM gene was detected in any isolate. The co-existence of two carbapenemase genes (NDM-1 + OXA) was seen in 12 (24%) isolates. Two isolates, one each from ST-22 and ST-15, were negative for both the OXA and NDM-1 genes. The CTX-M-15 gene allele was the major extended-spectrum β-lactamase (ESBL), with gene positivity in 49 isolates (98%). Details are given in Table 3. ST-14 and ST-15 were the predominant clones of the study population. The phylogenetic relationship of these isolates is shown in Figure 1.

**Table 3:** Resistance genes profile

Lab #	Date	Source	ESBL	Amp	Pip/Taz	Amik	Gent	Ceftr	Cefep	Imip	Mero	Cipro	Nitrof	TSX	Tige
231	6/1/2015	resp	N	32R	128R	64R	1S	64R	64R	16R	16R	4R	256R	320R	8R
232	10/1/2015	urine	N	32R	128R	64R	16R	64R	64R	16R	16R	4R	128R	320R	1S
233	13/1/2015	resp	N	32R	128R	64R	1S	64R	2I	8R	2I	1S	128R	20S	4I
234	18/1/2015	wound	NA	32R	128R	2S	1S	NA	64R	16R	16R	4R	NA	20S	4I
235	23/01/2015	wound	N	32R	128R	32I	16R	64R	64R	8R	8R	4R	128R	320R	8R
236	23/01/2015	wound	NA	32R	128R	64R	16R	NA	64R	16R	16R	4R	NA	320R	4I
237	25/01/2015	resp	N	32R	128R	2S	1S	64R	8I	2I	1I	0.25S	64I	20S	1S
238	26/02/2015	urine	NA	32R	128R	64R	1S	NA	64R	16R	16R	4R	NA	320R	8R
239	26/02/2015	urine	N	32R	128R	64R	16R	64R	64R	16R	16R	4R	256R	320R	8R
240	8/2/2015	resp	NA	32R	128R	64R	1S	NA	16I	16R	1I	4R	NA	320R	4I
241	12/2/2015	qc	P	32R	128R	2S	16R	64R	16I	2I	1I	4R	128R	320R	8R
242	12/2/2015	resp	N	32R	128R	64R	16R	64R	8I	16R	1I	4R	128R	320R	4I
243	12/2/2015	wound	P	32R	128R	64R	1S	64R	2I	16R	16R	0.25S	64I	60S	1S
244	21/2/2015	resp	NA	32R	128R	64R	16R	NA	64R	16R	1I	4R	NA	320R	4I
245	10/3/2015	resp	N	32R	128R	2S	1S	64R	4I	16R	1I	0.25S	64I	20S	0.5S
246	2/4/2015	blood	N	32R	128R	64R	1S	64R	64R	16R	1I	4R	64I	320R	4I
247	17/3/2015	resp	N	32R	128R	64R	16R	64R	64R	16R	16R	4R	256R	320R	8R
248	27/3/2015	wound	N	32R	128R	64R	16R	64R	64R	16R	16R	4R	256R	320R	8R
249	12/3/2015	resp	N	32R	128R	64R	4I	64R	64R	16R	16R	4R	512R	320R	8R
250	15/4/2015	urine	N	32R	128R	16S	1S	64R	64R	16R	16R	4R	256R	40S	8R
251	18/4/2015	wound	N	32R	128R	2S	1S	64R	8I	8R	8R	4R	256R	20S	8R
252	21/4/2015	urine	N	32R	128R	2S	1S	64R	2I	2I	1I	0.25S	64I	20S	0.5S
253	22/4/2015	blood	NA	32R	128R	2S	16R	NA	2I	2I	4R	2R	NA	320R	2S
254	27/4/2015	blood	N	32R	128R	2S	16R	64R	64R	8R	4R	4R	256R	320R	8R
255	29/4/2015	blood	P	32R	128R	64R	16R	64R	8I	16R	1I	1S	128R	320R	0.5S
256	1/5/2015	wound	N	32R	128R	16S	2S	64R	64R	16R	16R	0.5S	64I	20S	1S
257	6/5/2015	blood	N	32R	128R	64R	16R	64R	64R	4R	16R	4R	512R	20S	2S
258	5/5/2015	blood	N	32R	128R	64R	1S	64R	64R	16R	16R	4R	256R	320R	4I
259	9/5/2015	urine	P	32R	128R	64R	16R	64R	16I	4R	1I	4R	256R	320R	8R
260	12/5/2015	wound	NA	32R	128R	64R	16R	64R	64R	16R	16R	4R	256R	320R	8R
261	15/5/2015	urine	N	32R	128R	64R	4I	64R	64R	16R	16R	4R	256R	320R	8R
262	17/5/2015	urine	N	32R	128R	32I	1S	64R	64R	2I	16R	4R	256R	320R	8R
263	13/5/2015	wound	P	32R	128R	64R	1S	64R	2I	2I	0.5 I	0.25 S	64I	20S	0.5S
264	22/5/2015	other	NA	32R	128R	2S	1S	NA	64R	16R	16R	4R	NA	40S	4I
265	10/5/2015	blood	N	32R	128R	64R	8I	64R	64R	16R	16R	4R	256R	320R	8R
266	13/5/2015	blood	P	32R	128R	64R	1S	64R	2I	2I	1I	0.25S	64I	20S	0.5S
267	30/5/2015	urine	P	32R	128R	16S	1S	64R	8I	2I	1I	4R	128R	40S	2S
268	3/6/2015	urine	N	32R	128R	64R	16R	64R	64R	16R	16R	4R	256R	320R	8R
269	10/6/2015	blood	N	32R	128R	64R	16R	64R	64R	16R	16R	4R	256R	320R	8R
270	12/6/2015	resp	P	32R	128R	2S	1S	64R	2I	16R	1I	0.25S	32S	320R	1S
271	11/6/2015	blood	NA	32R	128R	2S	1S	NA	64R	16R	16R	4R	NA	20S	8R
272	17/6/2015	wound	NA	32R	128R	16S	16R	NA	64R	2I	1I	4R	NA	320R	4I
273	18/6/2015	blood	N	32R	128R	2S	1S	64R	64R	16R	16R	4R	256R	20S	4I
274	19/6/2015	blood	N	32R	128R	16S	16R	64R	64R	16R	16R	4R	64I	320R	2S
275	1/7/2015	blood	P	32R	128R	64R	16R	64R	8I	2I	1I	2R	64I	320R	2S
276	1/7/2015	urine	N	32R	128R	64R	1S	64R	16I	2I	1I	4R	128R	40S	8R
277	3/7/2015	urine	N	32R	128R	64R	16R	64R	64R	16R	16R	4R	512R	320R	8R
278	3/7/2015	blood	P	32R	128R	64R	1S	64R	4I	2I	1I	4R	128R	320R	8R
279	3/7/2015	urine	N	32R	128R	2S	1S	64R	16I	2I	1I	4R	128R	20S	8R
280	5/7/2015	blood	N	32R	128R	64R	16R	64R	64R	16R	16R	4R	256R	320R	8R

## Discussion

Multi-drug resistance among KP has become a world phenomenon, nullifying the benefits of high-profile clinical skills such as open-heart and transplantation surgeries [11]. Several reports have projected Saudi Arabia as a growing pool for such multi-drug resistant pathogens because of several reasons [11-13]. In addition to common ESBLs, the incidence of CTX-M and other carbapenems are on the increase [14]. Here, we report that all our isolates were positive for CTX-M-15, underlying the multi-drug resistant profiles of these isolates. CTX-M-15 has previously been reported to be dominant among KP isolates from Saudi Arabia and has also been reported among outbreak isolates elsewhere, where 55% of Klebsiella pneumoniae isolates showed the phenotypic characterization of high ESBL [14-15]. The Klebsiella pneumoniae-producing ESBL were positive for the SHV, TEM, and CTX-M β-lactamase genes; the CTX-M genes that were more prevalent were CTX-M-1 and CTX-M-9-like genes [14-15]. CTX-M-15 was present in isolates from all STs, indicating its epidemicity. The OXA-48 gene has previously been reported in MDR-KP isolates from National Guard Health Affairs (NGHA), Riyadh. Our present study also showed the presence of OXA-505 (a member of the OXA-48 family) in as many as 70% of studied isolates. OXA-232 is reported for the first time in our KP isolates [6,8,11]. These findings need further investigations in a larger number of samples. The phylogenetic relationship between those two phenotypes shows clear isolation between these two groups (Figure 1).

The incidence of NDM in as much as 32% of isolates is in agreement with the previous reports from Saudi Arabia [8,11,16]. The coexistence of OXA and NDM genes also has been previously reported and underpins the elevated MIC levels of Carbapenem for these isolates. None of the resistance genes is clone specific and evenly distributed among all sequence types of KP (Table 1).

A polyclonal KP population was observed with 12 different STs. ST-14, ST-29, and ST-15 were the predominant clones of our study population. Most of these STs are frequently reported from different parts of the globe [17,18]. Thirty-seven isolates formed four clonal complexes based on similarities in four or more loci out of the seven loci of the MLST scheme. ST-14 and ST-15 are single locus variants (SLV) of each other differing in one locus. ST-14, ST-15, and ST-29 have been previously reported from this region. ST-14 is now being considered as an endemic clone for colistin resistance [19]. The large numbers of certain sequence types might be due to their clonal expansion [20]. Genetic diversity and population structure have inferences, which have been drawn by the MLST tool. This study has shown several genetically related antibiotic-resistant KP STs grouped into at least four clonal complexes based on mono-, di-, and tri-locus variations and are designated as C1-C4 for our study (Table 2). In members of complex C1, there is a single locus variation in infB among their isolates, whereas in complex C2 and C3, there is a tri-locus variation involving the mdh, pgi, phoE, and tonB genes. Finally, complex C4 has a di-locus variation in pgi mad tonB genes. As we see, three complexes have locus variations in tonB, which are C2, C3, and C4.

The main limitation of this study is that these results might not give the true picture of antibiotic resistance in the whole country. A multi-centric study involving a larger number of isolates is needed to reach a meaningful conclusion and a true picture of antibiotic resistance in the Kingdom. Investigations on the plasmids carrying the resistance genes would also shed light on the horizontal transfer of these resistant genes. However, our study forms a base for such investigations.

## Conclusions

The study has revealed the presence of CTX-M-15 in almost all isolates. The study also shows the presence of carbapenemase genes, such as OXA and NDM, singularly or in combination. Newer OXA phenotypes, such as OXA-232 and OXA-505, were detected. Multilocus sequence typing revealed the incidence of a polyclonal KP pool, ST14 and ST15 being the predominant ones. Further studies should be focused on these lines to generate comprehensive data to form a solid database of an antibiotic-resistant gene profile among the bacterial pathogens in Saudi Arabia.
